# A randomised controlled feasibility trial comparing culturally adapted psychoeducation and treatment as usual for persons with bipolar disorders in Nigeria

**DOI:** 10.1192/bjo.2025.66

**Published:** 2025-06-27

**Authors:** Dung Ezekiel Jidong, M. Ishrat Husain, Tarela Juliet Ike, Ameer Khoso, Maigari Yusufu Taru, Charles Nnaemeka Nwoga, Christopher Francis, Shadrack B. Mwankon, John Ezekiel Jidong, Juliet Y. Pwajok, Suleman Shakoor, Atta Muhammad Asif, Siqi Xue, Nusrat Husain

**Affiliations:** Division of Psychology and Mental Health, University of Manchester, UK; Campbell Family Mental Health Research Institute, Centre for Addiction and Mental Health (CAMH), University of Toronto, Canada; Department of Sociology and Criminology, Teesside University, UK; Pakistan Institute of Living and Learning, Karachi, Pakistan; Department of Psychiatry, Jos University Teaching Hospital, Jos, Nigeria; Department of Psychology, University of Jos, Nigeria; Department of Sociology, Coal City University, Nigeria; Global Centre for Research on Mental Health Inequalities, Mersey Care NHS Foundation Trust, Liverpool, UK

**Keywords:** Bipolar disorder, cultural adaptation, psychoeducation, intervention, Nigeria

## Abstract

**Background:**

Bipolar disorders are a major cause of disability worldwide, with most of the disease burden attributed to those in low- and middle-income countries, including Nigeria. There is limited evidence on culturally appropriate interventions for bipolar disorders in Nigeria.

**Aims:**

The study aims to examine the feasibility, and acceptability of culturally adapted psychoeducation (CaPE) for treating bipolar disorders.

**Method:**

A randomised controlled trial (RCT) compared CaPE plus treatment as usual (TAU) with TAU alone among 34 persons with bipolar disorders in Jos, Nigeria. CaPE comprised 12 group sessions of in-person psychoeducation lasting approximately 90 min each, delivered on a weekly basis by clinical researchers supervised by clinical psychologists and consultant psychiatrists. The primary outcome was feasibility, measured by participants’ recruitment and retention rates. Other outcomes included acceptability as measured by the Service Satisfaction Scale (SSS), Brief Bipolar Disorder Symptom Scale (BBDSS), Patient Health Questionnaire (PHQ-9) and Quality-of-Life scale (EQ5D). Outcomes were assessed at baseline and weeks 12 and 24. Focus group (*n* = 10) and individual interviews (*n* = 5) were conducted with the CaPE + TAU group, recorded, transcribed verbatim and analysed using interpretative phenomenological analysis.

**Results:**

The CaPE+TAU group (*n* = 17) recorded a high participant recruitment and retention rate of 86% across 12 sessions, and also recorded a higher level of satisfaction with SSS compared with the TAU alone group; 87.5% indicated very satisfied compared with 66.7% indicated not sure in the TAU group. In terms of clinical outcomes, for PHQ-9 scores the intervention group showed a reduction from baseline to end of intervention (EOI) and follow-up, with differences of −12.01 and −7.39, respectively (both *P* < 0.001). The EQ5D index showed a notable improvement in the intervention group at both EOI and follow-up (*P* < 0.001). Lastly, BBDS scores decreased significantly in the CaPE+TAU group at both EOI and follow-up, with differences of −21.45 and −15.76 (both *P* < 0.001).

**Conclusions:**

The RCT of CaPE is a feasible, acceptable and culturally appropriate treatment option for bipolar disorders in Nigeria. Further adequately powered RCTs evaluating the intervention’s clinical and cost-effectiveness are warranted.

## Background

Bipolar disorders are chronic mental health problems that result in functional impairment and contribute to a significant disease burden worldwide.^1^ About 40 million people (0.53% of the global population), or 1 in 150 adults, are living with bipolar disorders.^2^ Bipolar disorders account for 7% of all disability-adjusted life years (DALY) caused by mental disorders.^3^ Persons with bipolar disorders tend to have higher rates of suicide mortality.^4,5^ Within the last decade, these mortality rates have substantially increased,^6^ suggesting the need for more targeted research to address the unresolved needs of persons with bipolar disorders. A recent meta-analysis found that, compared with the general population, persons with bipolar disorders have reduced life expectancy with about 13 years of potential life loss.^7^ Bipolar disorders are historically under-researched compared with other mental disorders, especially in sub-Saharan Africa and Nigeria.^8^ In Nigeria, the prevalence of bipolar disorders is estimated to be around 1.83% of the population of >230 million,^9^ amounting to about 5 million Nigerians suffering from bipolar disorders. However, due to limited evidence-based data, Jidong et al^8^ critiqued that there is little or no reliable information on the context-specific understanding of the prevalence, aetiology, diagnosis and treatment of bipolar disorders in Nigeria. Therefore, bipolar disorders’ current prevalence and suffering in Nigeria are far beyond what are captured in the above literature, and require urgent interventions.

Evidence suggests that interventions such as psychoeducation, family-focused therapy (FFT), cognitive–behavioural therapy (CBT) and interpersonal and social rhythm therapy (IPSRT) are helpful treatment options for bipolar disorders.^1^ Among these, psychoeducation is recommended as the first choice of psychological intervention for bipolar disorders.^10,11^ Well-structured psychoeducation programmes provide comprehensive information to patients, their families and/or caregivers. The content may include information about the nature of bipolar disorders; medications and their side-effects; potential triggering factors; recognition of early recurrence and symptom management strategies, stigma and discrimination; avoidance of substance use; and the importance of living a well-structured and productive lifestyle.^1,12^ Psychoeducation improves treatment adherence and reduces the risk of relapse and consequent hospitalisation.^1^ Psychoeducation further encourages persons with bipolar disorders to take ownership of their treatment and become active participants in their recovery strategies.^13^

The World Health Organization’s (WHO’s) Mental Health Gap Action Programme (mhGAP) Intervention Guide now recommends that psychoeducation be routinely offered for treatment of bipolar disorders in low- and middle-income countries (LMICs),^14^ and culturally adapted psychoeducation (CaPE) could be crucial in bridging the bipolar disorders treatment gap in Nigeria and other LMICs.^1,8^ CaPE can be co-designed and delivered in either an individual or group setting, and its proponents argue that it is a relatively user-friendly and cost-effective intervention.^11,15^ A previous study of CaPE in another LMIC, Pakistan, was shown to be acceptable, feasible and effective for treating bipolar disorders.^1^ However, CaPE needs to be culturally adapted and tested within the Nigerian culture and context.

Context and cultural specificity are essential in the aetiology, diagnosis and treatment of mental disorders.^16,17^ Inappropriate consideration of context-specific aetiology may lead to misdiagnosis and, consequently, incorrect or ineffective treatment.^18^ Considering the Nigerian context, embracing the Afrocentric ideology, also known as Afrocentrism, is essential to deconstruct the dominance of the Eurocentric model in Nigeria’s mental healthcare provision. Afrocentrism is keen on the renaissance of essential African cultural values and mental healthcare traditions previously discredited by Eurocentric ideologies through slavery, colonisation and neo-colonialism.^18,19^ There are numerous benefits of using the Afrocentric framework to underpin the CaPE intervention for persons with bipolar disorders. This is because Afrocentrism centres the experiences and perspectives of African people, promotes cultural affirmation and empowers collective responsibilities by highlighting their strengths and resilience, expressed through their Indigenous languages, cultural beliefs and traditional practices.

Afrocentrism is central to cultural adaption, and Nigerian cultural values and traditions are at the heart of planning and developing the intervention materials, such as the CaPE manual content and research protocols. For example, our preliminary study^8^ engaged with Nigerian persons with bipolar disorders, their family caregivers, clinicians and community members. Context-specific factors, including Nigerian cultural and religious beliefs, indigenous languages, literacy and service user preferences, were considered essential for psychological intervention. These factors were particularly helpful in understanding context-specific perspectives on bipolar disorders, including cultural idioms of mental distress, its perceived causes, including religious and language implications for potential interventions.^8,20^ Our previous work provided insight into contextual knowledge and cultural beliefs about bipolar disorders, informing the development and feasibility testing of a culturally adapted psychoeducation intervention for Nigerian persons with bipolar disorders. The present study evaluated the feasibility, acceptability and preliminary clinical effectiveness of CaPE in persons with bipolar disorders in Nigeria.

## Method

### Design

This study adopted a feasibility randomised controlled trial (RCT) design comparing CaPE + TAU with TAU alone. RCTs are highly commendable for testing psychological interventions due to their methodological rigour.

### Ethics approval and consent to participate

The authors assert that all procedures contributing to this work comply with the ethical standards of the relevant national and institutional committees on human experimentation, and with the Helsinki Declaration of 1975 as revised in 2013. All procedures involving human subjects/patients were approved by Jos University Teaching Hospital (no. JUTH/DCS/REC/127/XXXI/350). In addition, the study’s protocol was registered on ClinicalTrial.Gov (no. NCT05721196) on 22 March 2023. All ethical guidelines for conducting clinical trials and safeguarding were also observed. For example, participants provided written consent before participating in the study. All participation in the trial was entirely voluntary. Also, all participants were made aware of their right to withdraw their participation at any stage without being disadvantaged in any way.

### Study procedures

Participants were recruited from Jos University Teaching Hospital, Nigeria through referral and purposive sampling techniques. Patients were first recruited into the study on 27 March 2023. In total, 34 adults were recruited and randomly assigned to either CaPE + TAU (*n* = 17) or TAU alone (*n* = 17). This was carried out to ensure that, even following loss to follow-up, we would have at least 12 subjects per group for analysis. Recommendations regarding sample size were informed from prior studies, which suggested between 24 and 50 participants for a pilot or feasibility trials.^21,22^

### Eligibility criteria

The inclusion criteria were: diagnosis of DSM-5 bipolar disorders (type I or II), screened with Beck Depression Inventory (BDI <35) and Young Mania Rating Scale (YMRS <30); currently euthymic or non-euthymic; age 18–60 years; engaged with the mental health services for the preceding 6 months; able to give written informed consent; resident in the trial catchment area; and the ability to speak English.

The exclusion criteria were: severe cognitive impairment; currently experiencing relapse from chronic mania, hypomania, mixed or depressive episode; actively suicidal; and the presence of any chronic comorbid psychiatric disorders according to DSM-5 criteria, including other mental disorders such as chronic anxiety disorders, attention-deficit/hyperactivity disorder, impulse control disorders, substance use disorders or physical diseases such as chronic thyroid illness, obesity, type II diabetes and cardiovascular diseases.

### Randomisation

**Fig. 1:**
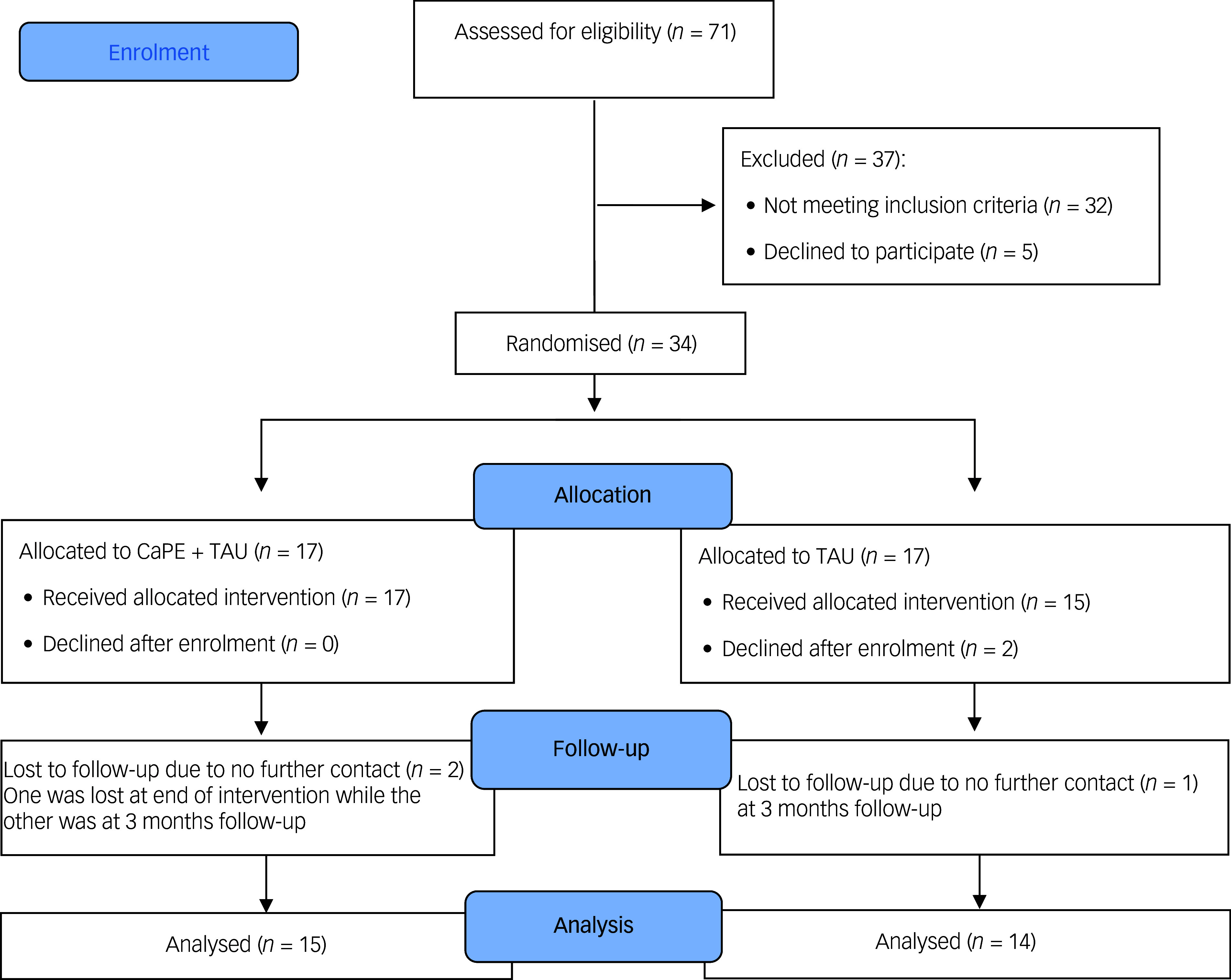
CONSORT flow diagram. CaPE, psychoeducation; TAU, treatment as usual; CONSORT, Consolidated Standards of Reporting Trials.

Randomisation was generated using the Microsoft Excel RANDBETWEEN function. All participants were assigned in a 1:1 ratio to either CaPE + TAU or TAU alone.^23^ All participants were blinded to whether they were assigned to the experimental (CaPE + TAU) or controlled (TAU alone) group (see Fig. 1). However, the feasibility trial was unable to double-mask the clinical researchers who delivered CaPE, due to their involvement in the intervention manual and study protocol co-adaptation and refinement.

### Cultural adaptation

The study adopted the Iterative Model of Co-adaptation (IMC),^16,20^ to ensure that the CaPE manual and study protocol were culturally appropriate and suitable for Nigerian persons with bipolar disorders. The IMC process considered context-specific factors such as cultural views about bipolar disorders (idioms of mental health distress, its perceived causes and Nigerian indigenous solutions). Furthermore, language and religious implications and the type or level of support participants needed for the CaPE intervention were also considered. All participants’ feedback was used to inform the CaPE manual’s content and practical utility.

### Intervention

The CaPE intervention consisted of 12 group sessions of psychoeducation. Each session lasted about 90 min on a weekly basis and was delivered alongside the TAU alone group. Group-based CaPE for bipolar disorders has been recommended.^11,15^ The rationale behind CaPE’s 12-session structure (see Table 1) is to provide participants with a smooth transition from learning about bipolar disorders and the subsequent treatment transformation. For example, each CaPE session began with a presentation on the day’s topic, followed by a related exercise (e.g. drawing a life chart or compiling a list of potential triggers for relapse and how these could be managed). The CaPE manual content was a modified version of the Barcelona Psychoeducation Programme for bipolar disorders^12^ used in Pakistan.^1^ However, illustrative examples of CaPE content were informed by our study team’s prior qualitative research, and further contextualised to reflect the Nigerian indigenous language of expressions, cultural values and religious beliefs in the weekly sessions, thereby valuing participants’ contributions in shaping the content of each session. CaPE contents and materials were delivered in English. However, scenarios, case examples and symptoms of relevant psychological disorders were explained in local languages by bilingual clinical researchers delivering the CaPE intervention.

**Table 1:** Participants’ attendance, retention and adherence to each CaPE intervention session

Sessions	CaPE + TAU *N = 17*	
	*n*	%
1. Concept and causes	15	88
2. Symptoms: mania, hypomania, depression and mixed conditions	15	88
3. Evolution and prognosis, psychoactive substance misuse	14	82
4. Treatment with medication (mood stabilisers, antipsychotics, antidepressants)	14	82
5. Alternative therapies	15	88
6. Risks associated with interruption of treatment	15	88
7. Learning to detect early symptoms of relapse	15	88
8. What to do when a relapse is detected	15	88
9. Regularity of habits	14	82
10. Stress control	15	88
11. Problem-solving strategies	13	76
12. Final session	15	88
Percentage ÷ 12 sessions, total		86

CaPE, culturally adapted psychoeducation; TAU, treatment as usual; *N*, total number of participants randomised to the CaPE intervention arm; *n*, total number of participants who attended each session; the percentage comprises those who attended out of the total 17 participants.

TAU in Nigeria consists of routine treatment comprising out-patient psychiatric care, including psychotropic medications and other psychological treatments such as cognitive behaviour or interpersonal psychotherapies for bipolar disorders, which are different from CaPE.

### Clinical researchers’ training and supervision

Two clinical researchers (SBM and CF) delivered the 12 sessions of CaPE intervention and were supervised weekly by *n* = 2 clinical psychologists (DEJ and JYP) and *n* = 2 consultant psychiatrists (MYT and CNN). The clinical researchers are bilingual, and both have above Master’s degree-level clinical psychology certification. They received additional 1-day (7-h) training on the CaPE manual and study protocol. Clinical researchers previously received 4 days (32 h) of training in delivering psychosocial interventions to adults with severe mental illness, including bipolar disorders.

To further ensure fidelity to the intervention manual for each CaPE session, clinical researchers conducted training on each session delivery with simulated patient cohort. They received feedback from senior clinical practitioners on a weekly basis prior to delivery of the actual session. The use of clinical researchers aligns with WHO recommendations on task shifting and the use of non-clinical experts in tackling workforce shortages in low-resource settings, such as Nigeria.^20,24^

### Outcome measures and assessment

The study’s primary outcome measure was to test the feasibility of recruitment, retention and acceptability of the CaPE intervention, which was assessed using recruitment rate, data on each session attendance, trial retention and the Service Satisfaction Scale (SSS)^25^ (see Table 1). The study’s secondary outcomes were assessed using the Brief Bipolar Symptom Scale (BBDS),^26^ Patient Health Questionnaire (PHQ-9)^27^ for depression and EQ5D for quality of life.^28^ Changes were assessed at various time frames, including baseline, at 12 weeks (end of the intervention, EOI) and 3 months post-intervention.

### Analysis

Quantitative data and primary outcome measures were analysed using the recruitment, attendance and dropout rates (see Table 1), including participant satisfaction with the intervention using SSS. Given the study’s feasibility nature, the small sample size did not allow for sufficient power to reject null hypotheses (see Fig. 1). However, inferential statistics was conducted based on the intention-to-treat principle. Analysis of covariance models were used to analyse continuous outcomes, in which the mean group difference was adjusted for baseline outcome scores.^1^ Binary outcomes were analysed using chi-square or Fisher’s exact test. If the expected values were small, these were used to analyse the binary outcomes. We used a two-sided approach for all statistical analyses and a confidence interval level set at 95%.

Qualitative data were analysed using interpretative phenomenological analysis (IPA), as recommended by Smith.^29^ IPA is the most suitable qualitative method to explore participants’ experiences of receiving the CaPE intervention, and their views on its effectiveness in treating and alleviating the symptoms of their bipolar disorders. The analysis was also grounded in three IPA theoretical elements: phenomenology, hermeneutics and ideography.^29,30^ In this context, the phenomenon of interest is participants’ experiences of the CaPE intervention for bipolar disorders. Hermeneutics involved the researchers’ experiential roles in examining participants’ efforts within the data-set, because they made sense of their ideographic experiences that are specifically related to bipolar disorders and the CaPE intervention. The IPA theoretical elements underpinned the analytical commentaries, which followed each extract of qualitative data verbatim in the results section.

## Results

The sociodemographics of the sample are reported in Table 2. The study included 32 participants, with 17 in the intervention group and 15 in the control group.

**Table 2:** Sociodemographic characteristics of the sample (*N* = 32)

Parameters	CaPE + TAU *n* = 17	TAU alone *n* = 15	Total *N* = 32
Gender			
Male	10 (58.8%)	5 (33.3%)	17 (53.1%)
Female	7 (41.2%)	10 (66.7%)	15 (46.9%)
Age (years)			
18–30	5 (29.4%)	5 (33.3%)	10 (31.3%)
31–45	12 (70.6%)	7 (46.7%)	19 (59.4%)
>45	–	3 (20.0%)	3 (9.4%)
Education			
Primary	–	1 (6.7%)	1 (3.1%)
Secondary	10 (58.8%)	9 (60.0%)	19 (59.4%)
Tertiary	7 (41.2%)	5 (33.3%)	12 (37.5%)
Occupation			
Unemployed	7 (41.2%)	11 (73.3%)	18 (56.3%)
Employed	10 (58.8%)	4 (26.7%)	14 (43.8%)
State of origin			
North-west	2 (12.5%)	2 (13.3%)	4 (12.9%)
North-central	14 (87.5%)	10 (66.7%)	24 (77.4%)
South-east	–	2 (13.3%)	2 (6.5%)
South-west	–	1 (6.7%)	1 (3.2%)

CaPE, culturally adapted psychoeducation; TAU, treatment as usual.

The study’s primary outcome measure was to test the feasibility and acceptability of the intervention in terms of recruitment, retention, adherence and satisfaction rates.

Based on the data in Table 1, we observed high retention rates in the CaPE intervention; overall participation rate was 86%.

Overall, the CaPE + TAU group showed a higher level of satisfaction (see Table 3), with 93.8% satisfaction with the intervention. In comparison, 93.3% of the TAU alone group indicated they were unsure. As indicated by the data, 93.8% of participants in the CaPE + TAU group believed the CaPE intervention was effective. In comparison, 86.7% of participants in the TAU alone group believed they were not sure that the hospital-based treatment as usual was effective. In terms of usefulness and quality of the intervention, 87.5% in the CaPE + TAU group indicated that it was of high quality, while in the TAU alone group, 73.3% felt it was of low quality. Regarding recommending the CaPE intervention to others, 93.3% indicated ‘definitely yes’. In contrast, in the TAU alone group, 50% believed they could not recommend the intervention to others.

**Table 3:** Participants’ satisfaction with the intervention

		CaPE + TAU versus TAU alone									
		*n* = 15					*n* = 15				
		%					%				
Survey questions		0	1	2	3	4	0	1	2	3	4
1.	Please rate your satisfaction on the acceptability of the intervention 0, unacceptable; 1, slightly acceptable; 2, not sure; 3, slightly acceptable; 4, acceptable		6.3			93.8			93.3	6.7	
2.	Please rate your satisfaction with the effectiveness of the intervention 0, not at all effective; 1, ineffective; 2, not sure; 3, effective; 4, very effective		6.3			93.8		13.3	86.7		
3.	How would you rate the quality and usefulness of the content of the intervention 0, very low quality; 1, low quality; 2, not sure; 3, high quality; 4, very high quality		6.3		6.3	87.5	6.7	53.3	26.7	13.3	
4.	How would you rate the quality of the intervention? 0, worst approach; 1, less quality; 2, not sure; 3, high quality; 4, best approach				12.5	87.5		73.3	20	6.7	
5.	How would you rate your satisfaction with the intervention? 0, not satisfied at all; 1, not satisfied; 2, not sure; 3, satisfied; 4, very satisfied				12.5	87.5	6.7	26.7	66.7		
6.	Would you recommend the intervention to others? 0, definitely no; 1, no; 2, not sure; 3, yes; 4, definitely yes				6.7	93.3		50	50		

CaPE, culturally adapted psychoeducation; TAU, treatment as usual.

### Prevalence of depression at baseline, EOI and follow-up.

As shown in Table 4, at baseline, both groups (CaPE + TAU and TAU alone) showed moderately severe scores on depressive symptoms. However, by EOI, the CaPE group showed a substantial improvement, with 76.5% of participants experiencing reduced depression. In contrast, the TAU alone group showed moderate depression cases at 60%, with no severe cases reported. follow-up, the CaPE + TAU group maintained their improvement, with 80% reporting minimal depression and none in the severe category. In comparison, the TAU alone group showed varied results: 35.7% (5 out of 14) moderate, 28.6% (4 out of 14) mild and 21.4% moderately severe cases. Overall, the CaPE + TAU group showed significant and sustained improvements in reduced depressive symptoms from baseline through EOI and follow-up, while the TAU group showed less consistent and generally poorer outcomes.

**Table 4:** Prevalence of depression at baseline, end of intervention and follow-up

Parameter	Baseline		End of intervention		Follow-up	
Depression	CaPE + TAU	TAU	CaPE + TAU	TAU	CaPE + TAU	TAU
Minimal	–	–	13 (76.5%)	–	12 (80.0%)	2 (14.3%)
Mild	–	–	3 (17.6%)	2 (13.3%)	2 (13.3%)	4 (28.6%)
Moderate	–	2 (13.3%)	–	9 (60.0%)	–	5 (35.7%)
Moderately severe	4 (23.5%)	8 (53.3%)	–	4 (26.7%)	1 (6.7%)	3 (21.4%)
Severe	13 (76.5%)	5 (33.4%)	1 (5.9%)	–	–	–

CaPE, culturally adapted psychoeducation; TAU, treatment as usual.

Other secondary outcome measures of the study were to determine the preliminary effects of the CaPE + TAU intervention on improving knowledge and attitudes towards bipolar disorder, mood symptoms and quality of life. The results are shown in Table 5.

**Table 5:** Secondary outcomes (continuous scale)

Outcome	Time frame	CaPE + TAU		TAU alone		Difference^b^	*P*-value
		*n*	Mean (s.d.)	*n*	Mean (s.d.)	Mean (95% CI)	
PHQ-9^a^	Baseline	17	22.65 (3.39)	15	18.20 (3.59)		
	EOI	16	2.06 (2.43)	15	14.07 (2.84)	−12.01 (−13.96 to −10.05)	**<**0.001
	Follow-up	15	2.47 (4.81)	14	9.86 (5.20)	−7.39 (−11.22 to −3.56)	**<**0.001
EQ5D index	Baseline	17	0.10 (0.41)	15	0.20 (0.41)		
	EOI	16	0.81 (0.17)	15	0.35 (027)	0.45 (0.29 to 0.63)	**<**0.001
	Follow-up	15	0.95 (0.10)	14	0.35 (0.41)	0.59 (0.37 to 0.82)	**<**0.001
BBDS	Baseline	17	36.12 (5.61)	15	36.40 (6.34)		
	EOI	16	4.81 (3.76)	15	26.27 (3.17)	−21.45 (−24.02 to −18.89)	**<**0.001
	Follow-up	15	3.60 (3.68)	14	19.36 (8.26)	−15.76 (−20.57 to −10.94)	**<**0.001

CaPE, culturally adapted psychoeducation; TAU, treatment as usual; PHQ-9, Patient Health Questionnaire; BBDS, Brief Bipolar Disorder Scale; EQ5D, Quality of Life; EOI, end of intervention.

a. Summary statistics are the mean (standard deviation) in each group. b. The mean difference between groups was also reported as the outcome for CaPE + TAU minus that for TAU only.

As shown in Table 5, there were significant improvements in the CaPE + TAU group compared with the TAU alone group across several outcomes. For PHQ-9 scores, the intervention group showed a substantial reduction from baseline to EOI and follow-up, with differences of −12.01 and −7.39, respectively (both *P* < 0.001). The EQ5D index showed a notable improvement in the CaPE + TAU group at both EOI and follow-up (*P* < 0.001). Lastly, BBDS scores decreased significantly in the CaPE + TAU group at both EOI and follow-up, with differences of −21.45 and −15.76, respectively (*P* < 0.001).

### Qualitative findings

The focus group (*n* = 10) lasted 70 min, individual interviews (*n* = 5) lasted approximately 60 min each and were conducted with the CaPE + TAU group. The focus group and all individual interviews were recorded and transcribed verbatim for data analysis. The rationale for both focus group discussion and individual interviews was to complement the richness in data nuances on the CaPE intervention that could not be obtained via focus group or individual interviews alone. Data saturation was reached at nearly 70 min in the focus group discussion and the 5th individual interview, and thus no qualitative data were further required. Qualitative findings are presented here, using direct quotes from data transcripts and pseudonyms to support the following themes in the data-set.

### Theme 1: reduced stigma and improved sense of empowerment

A notable pattern in the data-set was that, before the CaPE intervention, participants were of the view that stigma constituted a major factor in limiting their mental health help-seeking behaviour. Stigma was construed from a negative perspective, including its impact on their families, who might also suffer because of the condition of the patient. Such stigma creates a sense of dissociation and isolation. The implication of this prevents affected persons from speaking out. Elaborating about this issue, one participant said:

‘Also, by bipolar, what make patients not to come out, is stigmatisation. They can even stigmatise your family, stigmatise you. Even when you are good, some people say you are not good, making it difficult for people to come out. But with this education, whether they stigmatise me or not, that is their business. Since I know the truth, I can live my life as a normal being’ (Noah, 39 years).

Another participant, while commenting on the issue, also said:

‘You see, before this [CaPE] programme, I felt I had a problem. And that makes me sometimes withdraw myself from people. And I do withdraw myself even to work. But now I’ve applied the knowledge- now I’ve applied the manuscript [CaPE service user guidance]. I’m a teacher by a profession. I’ve applied the manuscript. I tell myself, I can do it! I’m ready to work. And even the relationship between me and members of family, my siblings, my parents, have improved. Like now. I can even, I was staying with members of my family. Now I’m planning to start saving a lot, to take a good care of myself. So, you see, that is good to the intervention. So, I guess if they can bring another intervention that can make us to become advocates in the community, going from one community to our community, advocating about the needs to improve mental health, so that people who look at bipolar as something, as a sickness without a cure. So that people will be free and when we work hard, stigmatisation will be over. That’s my prayer’ (Maimuna, 20 years).

The preceding extract highlights a sense of poor quality of life prior to the CaPE intervention, due to dealing with the effect of suffering from bipolar disorders. Some terms, such as ‘So that people will be free and when we work hard, stigmatisation will be over’, indicate the negative consequences of how extended communities perceive those affected by bipolar disorder as cursed. As the extract also shows, isolation through withdrawal from the extant community, including their source of livelihood, becomes a means of escaping the perceived stigma ascribed by the community. However, following engagement with the CaPE intervention, the participant recounted being brave enough to live with the condition and have an improved sense of self, including strengthened relationships with family members.

### Theme 2: exploring culturally rooted interpretations of mental health, instilling confidence and a high sense of gratitude

A significant finding from the data-set was the perception of a deep sense of historically rooted interpretations of mental health, especially those attached to people suffering from bipolar disorders. Such historically cultural beliefs include ascribing bipolar disorder to some spiritual causes that stem from the family of the affected patient. Commenting on this, one participant said that:

‘The myths surrounding mental health or sickness, like African will say – they will say, it’s spirit of your father. Or there are many false information going on, and that makes people shy away from having the sickness [bipolar disorders]. But now I have come to understand that it is a problem from the brain. So, I have learned through this knowledge, it makes me to accept my fate, I accept it. And because I accept it, God gives me the grace to work hard to fight it [bipolar disorder]. Like me now, I’m planning to become an advocate of mental illness’ (Nonso, 23 years).

Another participant recounts accepting his condition due to engaging in the CaPE intervention. While initial stigma conjures a sense of fear, the participant undergoing the intervention enables a sense of mindset shift by being able to manage his condition. More specifically, another participant said:

‘In terms of healing process and recovering process. At the initial stage, people are afraid because of the stigma they experience about the illness. But when we came here, I am free. I can go anywhere to say yes – I can enter television studio and announce it. Nobody, it will not remove anything in my body. Because I know I am at eternal stage. I have recovered almost fully now. So, if they say it, is left for them. But to me, I thank God for the healing process. I thank God for the intermediate stage. I thank God for the psychologist intervention. For the intervention this psychologist brought and the people over there that are sponsoring it, God will bless you all. Thank you very much. We are grateful’ (David, 23 years).

As indicated in the preceding extract, being able to engage with the CaPE intervention was construed from a positive perspective. Terms such as ‘I thank God for the psychologist’s intervention’ indicate a sense of relief from the CaPE intervention. A plausible reason why the participant might have expressed a high sense of gratitude might be the relief experienced during session engagement with the intervention. Such feelings include being in a safe space without judgemental attributes such as stigma or discrimination. The empathy alongside the intervention context appears to foster a positive sense of belonging which, from the participant, enabled him to reconcile and acknowledge his condition – collectively facilitating a positive recovery process.

### Theme 3: increased adherence to medications and improved quality of life

Most participants reported improved quality of life and enhanced relationships based on their engagement with the CaPE intervention. Speaking about this, one participant said that:

‘This [CaPE] programme has helped me in a lot of ways. Because one of the psychologists [clinical researchers] taught us so many things. I don’t play with my drugs [medications] any more. Before I used to play and missing to take my drugs. But now I’m not playing with my drugs any more. And they also taught us relaxation techniques, which after coming back home from work, I normally do it before going to bed. So, it has helped me a lot. And I also want this intervention more, not that I’m not grateful. I’m grateful! I thank you for all that you have done for us to pass through this intervention’ (Noah, 39 years).

Another participant also said that:

‘I was just playing – I often don’t want to take my meditation. But when I came here, I discovered so many things. How I wish this programme should extend to other people so that they know how to manage their lives’ (Rita, 28 years).

As the extracts show, initially the participants appeared unaware of the importance of being consistent with their medications. Terms such as ‘Before I used to play and missing to take my drugs’ indicate the poor approach adopted to manage medication uptake for their condition. Such an approach implies that it risks exacerbating the conditions, which could impact their quality of life or their ability to engage meaningfully with their career/work, host communities or relationships with others. As the extracts shows, engaging with the CaPE intervention was construed from a positive perspective and its implications for their mental health and well-being.

### Theme 4: bipolar disorder and general mental health awareness creation

A significant theme across the data-set showed participants’ wishes to expand the intervention to aid public awareness of mental disorders and wider outreach programmes. Such expansion was portrayed as being highly useful, especially for those who might need to be made aware of the content of CaPE, for example, by adhering to prescribed medications. As one participant said:

‘For this bipolar sickness, we need government to introduce it to many people, this type of lectures [CaPE programme] to people that maybe they are just having this [bipolar disorder] sickness for the first time, so that they should know how to handle it. And even like me, if I could have this knowledge at the beginning, maybe I would have been free from this sickness, I would not have bought drugs. Because people used to take drugs [medications], and when they have seen maybe one year, they are okay, they will leave [stop the medication]. After some time, the sickness will come back again. Because the doctor has not told you that you should stop the medication. So, we need that information, maybe government should help and give this education to more people about this bipolar disorder sickness’ (Ladi, 27 years).

As the intervention came to completion, an emotional statement, commendation and prayers ensued; as one of the participants said:

‘Oh, I’d say I am going to miss this [CaPE sessions]. I’m going to miss this people [fellow participants and clinical researchers] because all that you have been telling us is helping me a lot. A big thanks to God and the people who are sponsoring this programme. I pray that God will bless you all’ (Kolade, 28 years).

As the preceding extract shows, there was a perceived sense of gratitude for having partaken in the CaPE intervention and its usefulness in improving the participants’ mental health. Such a positive experience of engaging in the CaPE programme was instrumental to the participant’s view on extending the intervention to others with bipolar disorders who might find it beneficial in improving their mental health and well-being.

## Discussion

Our study examined the feasibility, cultural appropriateness and acceptability of CaPE intervention for managing bipolar disorders in Nigeria. Findings suggest that CaPE intervention is feasible, culturally appropriate and acceptable for Nigerian persons with bipolar disorders, as demonstrated by the higher satisfaction scores in participants randomly assigned to CaPE + TAU compared with TAU alone. Participants in the CaPE + TAU group reported improved mood symptoms and medication adherence compared with those who were randomised to TAU alone. This study’s quantitative and qualitative findings both suggest that the CaPE intervention is feasible, culturally acceptable and appropriate in the Nigerian context. A plausible explanation for its utility could be attributed to the cultural relevance of CaPE intervention from an Afrocentric theoretical lens,^19^ thus depicting contextually appropriate content and sensitivity to the Nigerian indigenous cultural values and belief systems. The Afrocentric framework provided a more accurate understanding of the bipolar disorders impacting the Nigerian populations, and challenges Eurocentric biases in treatment approaches by offering a unique lens on the CaPE intervention and understanding of the findings through an African-centred worldview.

These findings are similar to the previous feasibility study of CaPE, which was reported to be feasible and acceptable for the management of bipolar disorders in Pakistan.^1^ Like the present study, CaPE was also effective in improving mood symptoms compared with TAU alone in this context.^1^ Pakistan and Nigeria are two sovereign nations in different regions of the world. Nevertheless, they are both former British colonies and are now republics in the Commonwealth of Nations, having the similar challenges of LMICs such as a limited mental health workforce. CaPE was found to be culturally appropriate and acceptable, partly because, like Pakistan, Nigeria is also a heterogeneous society divided by geographical, tribal and cultural diversity.^31^ They are both countries with collectivist cultures that enjoy rich cultural, multi-ethnic, traditional and religious values.

Furthermore, the qualitative findings of the present study suggest that the CaPE intervention enabled positive management of social stigma towards bipolar disorders while aiding the debunking of myths and instilling confidence in acknowledging the presence of mental health problems. These qualitative reports align with another study from our group that examined Nigerian cultural beliefs about mental disorders,^32^ including bipolar disorders, and recommended approaches to tackle the stigma associated with mental health help-seeking behaviours.^18^

There is a potential link between demographic information, such as age and unemployment, and bipolar disorders. In the present study, 50.4% were within the age range 31–45 years, which is a productive age, yet 56.3% were unemployed at the time of this study – this suggests that those in poverty and socioeconomically disadvantaged persons are likely to be predisposed to bipolar disorders. The present study’s perspective on the age and unemployment status of bipolar disorder persons is supported in the literature.^33^ However, the participants’ experiences of CaPE intervention could have been different if they had been a majority from an upper-middle class and gainfully employed, or perhaps with much younger/older demographics.^34^ Overall, the discussion acknowledges the limitation of the literature on cultural adaptation of psychosocial intervention for persons with bipolar disorders in low-resource settings such as Nigeria.

### Limitations and recommendations for future trials

The study was conducted at Jos University Teaching Hospital, a single centre, and therefore findings may not be generalisable across Nigeria. Due to the challenges of homogenising standard care in low-resource settings such as Nigeria, using TAU alone as a controlled group might have inflated the clinical effect of the CaPE intervention despite the small sample size. Future trials with larger sample sizes should consider active comparators, such as unstructured psychosocial support, to minimise the risk of bias. Another limitation is that the instruments used for data collection (i.e. BBDS, PHQ-9 and EQ5D scales) were not validated for the Nigerian context. Adherence to medications was captured only in the qualitative findings – we recommend that future trials use standardised scales to capture quantitative data on medication adherence. The present trial could not be double-masked, and therefore we recommend future trials to extend blinding to the treatment physicians and the outcome assessors of group assignment.

In addition, the present study did not control for confounding variables such as types of bipolar disorder (i.e. type I or type II) and any change in medication during the study. These bipolar disorder subtypes might vary significantly in terms of illness severity, clinical and social outcomes, uptake and response to treatments and personal disability, and such variations might have impacted our findings in numerous ways. We recommend that future trials control for these potential confounding variables. Finally, because the clinical measures were all based on self-report, they may be prone to biases, including social desirability and recall bias.

### Strengths and original contributions

This study makes an original contribution to the mental healthcare discipline using a methodologically rigorous randomised controlled feasibility trial design. Findings showed that the CaPE intervention is feasible, acceptable and culturally appropriate for treating bipolar disorders. The intervention recorded high recruitment and retention rates. The high retention rate could be attributed to the CaPE programme of activities, which was culturally, linguistically and religiously appropriate and drew on content, including scenarios the participants could relate to and the interactive nature of the CaPE sessions.

Finally, our study marks a significant milestone as the first randomised controlled feasibility trial to employ a culturally adapted psychological intervention for bipolar disorders in Jos, Nigeria. This pioneering approach can potentially bring about a significant and positive change in the Nigerian mental health sector. Furthermore, this feasibility trial has established new knowledge on how bipolar disorders management could be sustainably improved in low-resource settings such as Nigeria, by integrating Nigerian indigenous cultural values and religious beliefs relevant to the beneficiaries. This aligns with the Afrocentric theoretical lens,^19^ which utilises culturally appropriate mental healthcare.

### Implications for practice, policy and further research

This study’s implication for mental health practice is far-reaching because it combines principles of clinical psychology, health psychology, psychotherapy and psychiatry. For example, the CaPE intervention imparts knowledge while equipping persons with bipolar disorders with the necessary skills to manage the condition, thereby complementing clinical practice and mental healthcare service provision. The implications of CaPE extend beyond maintaining bipolar disorder remission in the affected patients and further shaping care provision, to potential relevance for policy initiatives.

The study has generated compelling data regarding its policy implications. To understand the policy landscape concerning CaPE intervention as a maintenance treatment option for bipolar disorders in Nigeria, the findings have been discussed in the context of knowledge translation and consultations with Nigerian members of parliament, government executives, mental health opinion leaders, clinicians, service users and community members. Although the current study is in its feasibility stage with limited evidence, it has established a foundation for future research wherein policymakers and other stakeholders will be at the forefront of the research activities from start to finish.

The present study’s implications for future research are promising. Our findings demonstrate the potential clinical and cost implications of CaPE intervention for treating bipolar disorders in low-resource settings such as Nigeria, and are substantially encouraging. Thus, the CaPE intervention will be further utilised in a fully powered trial to evaluate its clinical and cost-effectiveness. Thus, a fully powered trial of CaPE will enhance clinical psychology practice and mental health policy related to treating bipolar disorders in Nigeria and other low-resource settings, thus improving evidence-based scientific knowledge and literature.

Finally, the present study’s findings are relevant to the existing literature and knowledge transferability. For example, our CaPE study is relevant to the increasing yearning for culturally relevant mental healthcare around the globe,^1,8,16^ and the usefulness of task-shifting, especially in low-resource settings of low- and middle-income and high-income countries,^24^ respectively. Thus, the findings from the present study have motivated the testing of CaPE feasibility and acceptability among British African and Caribbean persons with bipolar disorders in the UK.

Bipolar disorders are among the leading causes of disability worldwide, especially in the absence of culturally appropriate care in low-resource settings such as Nigeria. Although pharmacological interventions are helpful, augmenting medication with psychosocial approaches enhances outcomes for persons with bipolar disorders. The present study adds to the evidence base for culturally appropriate and potentially scalable psychosocial interventions for bipolar disorders in LMICs, including Nigeria. CaPE intervention is a feasible, acceptable and culturally appropriate intervention with high satisfaction among Nigerian persons with bipolar disorders. A larger RCT is warranted, to evaluate the longer-term clinical and cost-effectiveness of CaPE in Nigeria.

## Data Availability

The paper reports all relevant data for this study. Due to ethical restrictions, no further supplementary data are available.

## Clinical trials registration

The trial was registered on ClinicalTrial.Gov (no. NCT05721196).
